# β-Arrestin1 Promotes Colorectal Cancer Metastasis Through GSK-3β/β-Catenin Signaling- Mediated Epithelial-to-Mesenchymal Transition

**DOI:** 10.3389/fcell.2021.650067

**Published:** 2021-04-28

**Authors:** Qing Song, Zhifen Han, Xinnan Wu, Yan Wang, Lihong Zhou, Liu Yang, Ningning Liu, Hua Sui, Jianfeng Cai, Qing Ji, Qi Li

**Affiliations:** ^1^Department of Medical Oncology and Cancer Institute of Integrative Medicine, Shuguang Hospital, Shanghai University of Traditional Chinese Medicine, Shanghai, China; ^2^Academy of Integrative Medicine, Shanghai University of Traditional Chinese Medicine, Shanghai, China; ^3^Department of Medical Oncology, Suzhou TCM Hospital Affiliated to Nanjing University of Chinese Medicine, Suzhou, China; ^4^Department of Chemistry, University of South Florida, Tampa, FL, United States

**Keywords:** colorectal cancer, epithelial-to-mesenchymal transition, metastasis, β-arrestin1, Wnt/β-catenin signaling pathway

## Abstract

Recurrence and metastasis seriously affects the prognosis of patients with tumors, and the epithelial-to-mesenchymal transition (EMT) plays a key role in promoting tumor invasion and metastasis. Previous studies have showed that β-arrestin1 acted as a tumor-promoting factor in multiple types of tumor. However, the exact role and mechanism of β-arrestin1 in colorectal cancer (CRC) progression remains to be elucidated. Our research aimed to explore the potential mechanism underlying the role of β-arrestin1 in CRC metastasis. The expression of β-arrestin1 was investigated in both primary and metastatic CRC tissues using the GSE41258 database, and it was revealed that CRC patients with liver/lung metastasis had a higher expression level of β-arrestin1, and the expression level of β-arrestin1 was inversely correlated with the prognosis of CRC patients. Further *in vitro* mechanism studies indicated that β-arrestin1 had the ability to promote the migration of CRC cells through regulating the EMT process by activating Wingless/integration-1 (Wnt)/β-catenin signaling pathways. Blocking Wnt/β-catenin signaling with inhibitor ICG001 decreased the promoting effect of β-arrestin1 on EMT in CRC. *In vivo* imaging experiments further demonstrated the promoting effect of β-arrestin1 on the lung metastasis of CRC cells by tail vein injection in mice. The results of this paper suggest that β-arrestin1 promotes EMT *via* Wnt/β-catenin signaling pathway in CRC metastasis, and provides a novel therapeutic target for CRC metastasis.

## Introduction

Colorectal cancer (CRC) is the second most frequent malignant cause of cancer-associated mortality worldwide, leading to ∼0.8 million new deaths per year (Bray et al., 2018). Metastasis is the main cause of treatment failure and poor prognosis (Brenner et al., 2014). Multiple signaling pathways and key factors are involved in the migratory process of CRC cells from the primary sites into the distal locations/organs (Song et al., 2018).

A series of results have shown that the epithelial-to-mesenchymal transition (EMT) acts a vital role in promoting tumor invasion and metastasis (Samy et al., 2014). During EMT, loss of a pical-basal polarity, reorganization of the cytoskeletal architecture and increased cell motility contribute to cancer cells acquiring invasive and metastatic ability (Nieto et al., 2016). Various important genes or proteins are involved in regulating EMT, such as E-cadherin, N-cadherin and Vimentin, etc. (Xia et al., 2018). Multiple signaling pathways, such as transforming growth factor-β (TGF-β)/SMAD, phosphatidylinositol 3-kinase (PI3K) and mitogen-activated protein kinase (MAPK), have been reported to regulate the EMT process (Derynck and Zhang, 2003; Mu et al., 2012; Zhang et al., 2018). Recently, several experiments have revealed that the Wnt signaling is closely associated with the EMT. However, the detailed mechanism in CRC remains unclarified (Nawshad et al., 2007; Sara et al., 2011; Duan et al., 2016; Liu et al., 2017).

β-arrestins, including β-arrestin1 and β-arrestin2 (arrestin-2 and -3, respectively), are important intracellular regulatory proteins, and both of which were initially shown to act as scaffold to regulate signal transduction mediated by G protein coupled receptor (GPCR) (Lee et al., 2014). β-arrestin1 (53 kDa), located on chromosome 7, is comprised of two major domains, the N-domain and C domain, which consists of β-sheets and connecting loops with one short α-helix (Han et al., 2001; Song et al., 2018). β-arrestin1 serves as ligand-regulated scaffold for GPCR-mediated signaling. A previous study has shown that blocking β-arrestin-dependent GPCR desensitization could decrease the occurrence of drug tachyphylaxis in chronic pain, asthma and psychiatric illness. As signaling scaffold, β-arrestin1 also participates in regulating cell proliferation, migration and survival in inflammatory diseases, fibrosis and cancers (Peterson et al., 2017). In particular, β-arrestin1 was capable of regulating cell proliferation, facilitating invasion and migration, inhibiting apoptosis and affecting other characteristics of tumors, such as tumor growth rate, angiogenesis, drug resistance in a variety of tumors, including lung cancer, breast cancer, colorectal cancer, prostate cancer and ovarian cancer, etc. (Ge et al., 2004; Rosanò et al., 2005; Lakshmikanthan et al., 2009; Kim et al., 2010). It has also previously been shown that β-arrestin1 plays an important role in the development and progression of CRC. A previous study revealed that prostaglandin E2 (PGE2)-induced transactivation of the epidermal growth factor receptor (EGFR) and downstream Akt signaling through β-arrestin1 acts as a mediator in GPCR activation of c-Src, thereby promoting CRC cell migration and metastasis (Buchanan et al., 2006). Recent research indicated that microRNA (miR)-296 inhibits CRC cell proliferation and enhances apoptosis by activation of Akt mediated by β-arrestin1 (Zhang et al., 2019). Another study revealed that β-arrestin1 could decrease E-cadherin expression and increase Vimentin expression in tumor EMT process (Duan et al., 2016). Although previous studies have suggested the role of β-arrestin1 in promoting EMT in CRC, the underlying molecular mechanisms of β-arrestin1 regulating EMT are largely unknown.

This article investigated the biological functions and molecular mechanism of β-arrestin1 in EMT and metastasis of CRC, with the aim of providing a potential therapeutic target for CRC progression.

## Materials and Methods

### Data Analysis Using the GSE41258 Dataset

The β-arrestin1 expression and clinical data from the GSE41258 dataset were collected, including CRC primary tissues, lung metastasis tissues and liver metastasis tissues. β-arrestin1 expression was measured using the IlluminaHiSeq platform. There were 206 samples, including 155 primary tumor, 32 liver metastasis and 19 lung metastasis tissues in the GSE41258 dataset. The influence of β-arrestin1 expression on survival was assessed using the Cox proportional hazards model. The genes interacting with β-arrestin1 were screened *via* Pathway Commons, Kyoto Encyclopedia of Genes and Genomes (KEGG), Gene Ontology (GO) and dbEMT.

### Cell Culture

Human CRC cell line HCT-116 [American Type Culture Collection (ATCC)] was cultured in RPMI 1,640 medium and LoVo (ATCC) was cultured in Ham’s F-12K medium, containing 10% fetal bovine serum (FBS), 100 U/ml penicillin, 100 mg/ml streptomycin, and incubated in a humidified, 5% CO_2_ atmosphere, at 37°C. HCT-116 or LoVo monoclonal cells stably expressing shRNA/β-arrestin1, empty vector or over/β-arrestin1, were generated by screening with purinomycin (2 μg/ml). Other human CRC cells including HCT-8, Caco-2 and RKO cells were cultured as HCT116 cells, and SW620 cells were cultured in Leibovitz’s L-15 medium supplemented with 10% FBS, 100 U/ml penicillin, 100 mg/ml streptomycin.

### Plasmid Construction and Lentivirus Packaging

A total of three short hairpin shRNAs for the β-arrestin1 gene were used in the research, the sequences for which were as follows: shRNA/β-arrestin1-1: 5′-GAA CTGCCCTTCACCCTAATG-3′; shRNA/β-arrestin1-2: 5′-TCT GGATAAGGAGATCTATTA-3′; shRNA/β-arrestin1-3: 5′-GGA TCATTGTTTCCTACAAAG-3′, and the control shRNA sequence was as follows: 5′-TTCTCCGAACGTGTCACGT-3′. Human β-arrestin1 (Gene ID: 6421) gene was chemically synthesized. Both the shRNA/β-arrestin1 sequence and the over/β-arrestin1 sequence were subcloned into lentiviral expression vector (PGMLV-CMV-MCS-EF1-ZsGreen1-T2A-Puro), obtained from Genomeditech Co. The shRNA lentivirus, the control lentivirus and the overexpression lentivirus were transfected into the relatively high expressing β-arrestin1 cell lines HCT-116 and LoVo. β-catenin signaling inhibitor ICG001 was purchased from APExBIO.

### Lentivirus Transfection

Prior to transfection, HCT-116 and LoVo cells (4 × 10^5^) were seeded in 6-well plates in an incubator at 37°C overnight. Then, the shRNA lentivirus, the control lentivirus and the overexpression lentivirus were added into the plates [Lentivirus volume = (MOI × number of cells)/lentivirus titer], and cultured in suitable medium. After 48 h of transfection, the medium containing lentivirus was removed and the fresh medium was added.

### Western Blotting

In brief, all of proteins were prepared according to the instructions of ProteoJET Cytoplasmic kit (Fermentas Life Sciences). BCA protein assay was used for protein quantification. Proteins were separated onto the 10% SDS-PAGE gels for electrophoresis, then the protein on the gel was transferred to PVDF membranes, blocked in 5% milk, and incubated with the primary antibodies. Rabbit monoclonal antibodies against human E-cadherin (cat. no. 3195; dilution 1:1,000), N-cadherin (cat. no. 13116; dilution 1:1,000), Vimentin (cat. no. 5741; dilution 1:1,000), Snail (cat. no. 3879; dilution 1:1,000), β-arrestin1 (cat. no. 12697; dilution 1:1,000), GSK-3β (cat. no. 12456; dilution 1:1,000), p-GSK-3β (cat. no. 5588; dilution 1:1,000), Axin1 (cat. no. 2087; dilution 1:1,000), CK1 (cat. no. 2655; dilution 1:1,000), β-catenin (cat. no. 8480; dilution 1:1,000), c-Myc (cat. no. 5605; dilution 1:1,000), CyclinD1 (cat. no. 2922; dilution 1:1,000) and PCNA (cat. no. 13110; dilution 1:1,000) were purchased from Cell Signaling Technology, Inc. Rabbit monoclonal antibodies against human MMP2 (cat. no. sc-13594; dilution 1:500) and MMP9 (cat. no. sc-393859; dilution 1:500) were purchased from Santa Cruz Biotechnology, Inc. Mouse monoclonal antibodies against human GAPDH (cat. no. 60004-1-Ig; 1:1,000) were purchased from ProteinTech Group, Inc. The horseradish peroxidase-conjugated goat anti-rabbit secondary antibody (cat. no. A0208; dilution 1:1,000) and horseradish peroxidase-conjugated goat anti-mouse secondary antibody (cat. no. A0216; dilution 1: 1000) were purchased from Beyotime Biotechnology, Inc.). Primary and secondary antibodies were incubated in room temperature for 2 h. All the results were visualized *via* enhanced chemiluminescence. Each experiment was repeated independently three times.

### Immunofluorescence Microscopy

Following transfection, HCT-116 and LoVo cells (3 × 10^4^) that adhered to the coverslips were fixed with 4% paraformaldehyde for half an hour at room temperature, then infiltrated with Triton X-100 (0.5%) for 15min, and subsequently blocked with 5% BSA solution. The cells were first stained with E-cadherin (cat. no. 3195; dilution 1:200; Cell Signaling Technology, Inc.) rabbit antibody followed by Cy3-conjugated goat anti-rabbit IgG (cat. no. P0183; dilution 1:1,000; Beyotime Biotechnology, Inc.), or first stained with the Vimentin (cat. no. 5741; dilution 1:200; Cell Signaling Technology, Inc.) rabbit antibody followed by FITC-conjugated goat anti-rabbit IgG (cat. no. P0186; dilution 1:1,000; Beyotime Biotechnology, Inc.). Primary antibodies were incubated in room temperature for 60 min. The second antibody was incubated in dark for 60 min. After incubating with first and secondary antibodies at room temperature, DAPI was applied for nuclear staining with 30 min. Finally, cells were tested with a DMI3000B inverted microscope (Leica Microsystems GmbH). All experiments were repeated three times.

### Transwell Assay

Following transfection, HCT-116 (5 × 10^4^ cells seeded in RPMI 1640 medium with 0.5% FBS) and LoVo cells (5 × 10^4^ cells seeded in Ham’s F-12K medium with 0.5% FBS) were seeded into the upper chamber of the Transwell assay. A total of 600 μl RPMI 1640 or Ham’s F-12K medium with 10 μg/ml fibronectin and 15% FBS was mixed in the lower chamber. The data were collected after co-culture for 48 h. Crystal violet staining was used to detect migrated cells, followed by observation under a DMI3000B inverted microscope (Leica Microsystems GmbH). A total of five fields were randomly selected to count the migrated cells. Each experiment was repeated independently three times.

### Wound Healing Assay

After transfection, HCT-116 or LoVo cells were seeded on 6-well plates, an artificial scratch wound was created using a 20 μl pipette tip, and detached cells were removed through washing with PBS three times. The percentage of serum used for the wound healing assay was 0.5%. After 48 or 72 h incubation, cell migration images were captured using an inverted microscope and evaluated by measuring the difference in wound width.

### Reverse Transcription PCR

The primers for the RT-PCR were as follows: β-arrestin1: Forward, 5′-ACCAGGCAGTTCCTCATGTC-3′; reverse, 5′-CG TCTTGTTGGTGTTGTTGG-3′; GAPDH: Forward, 5′-GAAGG CTGGGGCTCATTTG-3′; reverse, 5′-GGGCCATCCACAGT CTTC-3′. RT-PCR was performed using SYBR^®^ 2xTaq Master Mix (Vazyme Biotech Co., Ltd.) with an iCycler^®^ thermal cycler (Thermo Fisher Scientific, Inc.) All assays were performed in triplicate and independently repeated three times.

### Animal Model

Male BALB/c nude mice with five weeks were bought from the Animal Experimental Center of Shanghai University of Traditional Chinese Medicine and raised in specific pathogen-free laboratory animal room for a week. The mice were injected with 2 × 10^6^ HCT-116/luc cells, or shRNA/β-arrestin1-HCT-116/luc cells, or over/β-arrestin1-HCT-116/luc cells through the tail vein. For *in vivo* imaging, mice were anesthetized through intraperitoneal injection of 2% sodium pentobarbital (40 mg/kg) and injected intraperitoneally with D-luciferin (15 mg/ml in 200 μl PBS), and observed using an IVISLumina system (Caliper Life Sciences). The maximum tumor size in the mice following the injection of the HCT-116 cells did not exceed 1,000 mm^3^. Until the end of the animal experiments, all mice were euthanized by CO_2_ exposure according to the IACUC policy, and the optimal flow rate was set to 20% of the chamber volume per min until the mice were unconscious. Then, the excised lungs were fixed in 4% paraformaldehyde, and embedded in paraffin. Paraffin-embedded lung tissue were fully cut, and each section was 6 μm thick. All lung sections were stained with hematoxylin and Eosin (HE), and assessed for E-cadherin and Vimentin expression *via* immunohistochemistry. At the beginning, eight mice of each group were included, and each mouse was fed with water and food randomly and enabled to adapt to the environment for a week prior to formal experiment. The duration of the formal experiment was approximately 43 days. The mice were euthanized if displaying severe illness before the end of the formal experiments in order to keep the pain to a minimum. The health and behavior of mice were estimated twice a day. The criteria for deciding when to euthanize the animals including lack of feeding or drinking, weight loss (15–20%), ruffled fur, loss of appetite, weakness, symptoms of severe organ system dysfunction and no response to treatment. The care of the experimental animals and animal experimentation were carried out in accordance with animal ethics guidelines and permitted by the Animal Care and Use Committee at Shanghai University of Traditional Chinese Medicine, and the permit number of the approved animal work provided by the ethics committee was PZSHUTCM190308013. In addition, our animal experiments were approved in July 13th, 2018, and the animal experiments were performed between August 1st, 2018 and February 28th, 2019.

### Statistical Analysis

All data were presented as the mean ± SD of at least three independent experiments and analyzed with SPSS software (version 22). The mean values of two groups were compared by Student’s *t* test. Kaplan-Meier method was used to calculate the survival times and Log-rank test for the difference significance. ANOVA was used for multiple comparisons, and Tukey’s *post hoc* test was performed after ANOVA. *P* < 0.05 was considered to indicate a statistically significant difference.

## Results

### High β-Arrestin1 Expression Correlates With CRC Metastasis

**FIGURE 1:**
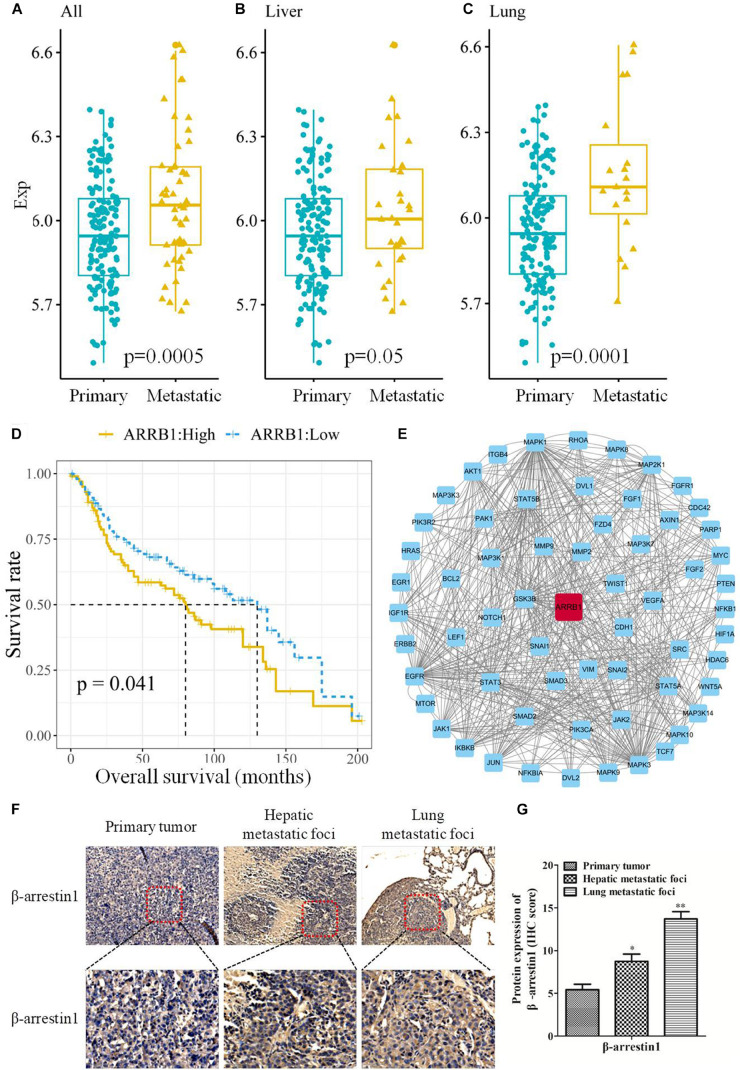
Expression of β-arrestin1 in CRC tissues and its association with the prognosis of patients with CRC. **(A–C)** Analysis of β-arrestin1 expression profile using data from the GSE41258 dataset. **(D)** Association between β-arrestin1 expression and the prognosis of patients with CRC. **(E)** Network representation of β-arrestin1 interactions with multiple genes, including Twist, Snail, CDH1, Vimentin, MMP2, MMP9, GSK-3β, MAPK, etc. **(F–G)** Immunohistochemical and quantitative analysis of β-arrestin1 protein in representative CRC and metastatic lung/liver tissues (scale bars, upper row: 200 μm, down row: 50 μm, respectively). *P < 0.05; **P < 0.01 vs. Primary tumor.

To study the correlation between β-arrestin1 and CRC metastasis, the expression of β-arrestin1 in CRC tissues was analyzed using the GSE41258 dataset. There were 206 samples in the dataset, including 155 primary tumor, 32 liver metastasis and 19 lung metastasis tissues. Through data analysis, it was revealed that β-arrestin1 was markedly overexpressed in CRC metastasis tissues compared with CRC primary tissues (*P* = 0.0005; Figure 1A). In particular, liver metastasis tissues had a higher level of β-arrestin1 than primary CRC tissues (*P* = 0.05; Figure 1B). Compared with CRC primary tissues, the expression of β-arrestin1 also significantly increased in lung metastasis tissues (*P* = 0.0001; Figure 1C). Accordingly, CRC patients with high expression levels of β-arrestin1 in CRC primary tissues had a poorer prognosis, compared with those with low expression levels of β-arrestin1 (*P* = 0.041; Figure 1D). Moreover, the 5-year and 10-year survival rates of CRC patients with low expression levels of β-arrestin1 in CRC primary tissues were 41.86% and 16.28%, in compared with 38.10% and 14.29% in CRC patients with high expression levels of β-arrestin1 in CRC primary tissues. Pathway Commons, KEGG, GO and dbEMT were used to analyze the genes co-expressed alongside β-arrestin1. The genes with degree ≥ 10 in the network were presented in Figure 1E. The results revealed that β-arrestin1 was closely associated with the genes of EMT, including Twist, Snail, CDH1, Vimentin, MMP2 and MMP9, and GSK-3β, NF-κB, PI3K/Akts, MAPK signaling pathways were involved in regulating this process (Figure 1E). Next, the immunohistochemistry staining further revealed significantly higher protein levels of β-arrestin1 in lung/liver metastatic foci than the ones in primary tumors (Figures 1F,G), implying that β-arrestin1 overexpression correlated closely with CRC metastasis.

### β-Arrestin1 Regulates EMT in CRC Cells

**FIGURE 2:**
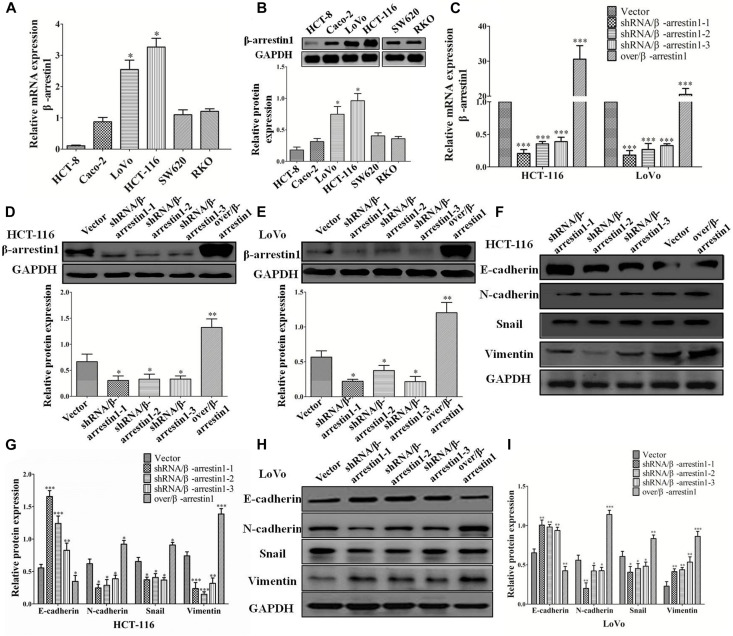
β-arrestin1 regulates the EMT of CRC cells. **(A,B)** The expression of β-arrestin1 was detected in six CRC cell lines by reverse transcription quantitative PCR. β-arrestin1 had a higher expression level in LoVo and HCT-116 cells, ^∗^*P* < 0.05 vs. the other four established human CRC cell lines HCT-8, Caco-2, SW620 and RKO. **(C)** The mRNA expression of β-arrestin1 in shRNA/β-arrestin1 and over/β-arrestin1 groups was detected by reverse transcription PCR. ^∗^*P* < 0.05; ^∗∗^*P* < 0.01 vs. HCT-116-vector or LoVo-vector cells. **(D–E)** Western blotting detected the protein expression of β-arrestin1 in shRNA/β-arrestin1 and over/β-arrestin1 groups in HCT-116 or LoVo cells. ^∗∗∗^*P* < 0.001 vs. HCT-116-vector or LoVo-vector cells. **(F–I)** Expression levels of epithelial or mesenchymal markers in HCT-116 or LoVo cells were analyzed by western blotting. ^∗^*P* < 0.05; ^∗∗^*P* < 0.01; ^∗∗∗^*P* < 0.001, vs. HCT-116-vector or LoVo-vector cells. EMT, epithelial-to-mesenchymal transition; CRC, colorectal cancer; shRNA, short hairpin RNA.

Since the bioinformatics analysis suggested a close association between β-arrestin1 and the EMT-associated signaling pathway, and EMT is a key step in the invasion and metastasis of CRC, next, we observed the function of β-arrestin1 in EMT. β-arrestin1 expression was characterized in six CRC cells. It was revealed that β-arrestin1 was highly expressed in HCT-116 and LoVo cells, compared with HCT-8, Caco-2, SW620, and RKO cells (Figures 2A,B). For the purpose of elaborating the function of β-arrestin1, we firstly knockdowned and overexpressed the β-arrestin1 gene in CRC cells. Through RT-PCR, the data confirmed that the expression of β-arrestin1 was markedly downregulated in shRNA/β-arrestin1 groups, and significantly upregulated in the over/β-arresin1 group, when compared with the control group (Figure 2C). The protein expression levels of β-arrestin1 in the aforementioned groups were also detected *via* western blotting, and the results were consistent with the RT-PCR results (Figures 2D,E).

Epithelial-to-mesenchymal transition is primarily characterized by the destruction of adherens junctions (AJs), which is due to cells losing epithelial characteristics (Yeung et al., 2005; Lakins et al., 2012). In the process, the conversion from E-cadherin to N-cadherin results in decreasing the function of AJs (Przybyla et al., 2016). The change of cytoskeletal and polarity complex proteins leads to EMT (Huang et al., 2012). The activation of Vimentin could regulate organelles and membrane-associated proteins, and promote cell motility (Mendez et al., 2010). Therefore, E-cadherin, N-cadherin, Vimentin and Snail are recognized broadly as the characteristic proteins of EMT. The data we observed indicated that, compared with the control groups, the expression of E-cadherin increased markedly, and the expression of Snail, N-cadherin and Vimentin markedly decreased in shRNA/β-arrestin1 groups, while the over/β-arrestin1 groups had an opposite effect in HCT-116 or LoVo cells (Figures 2F–I).

**FIGURE 3:**
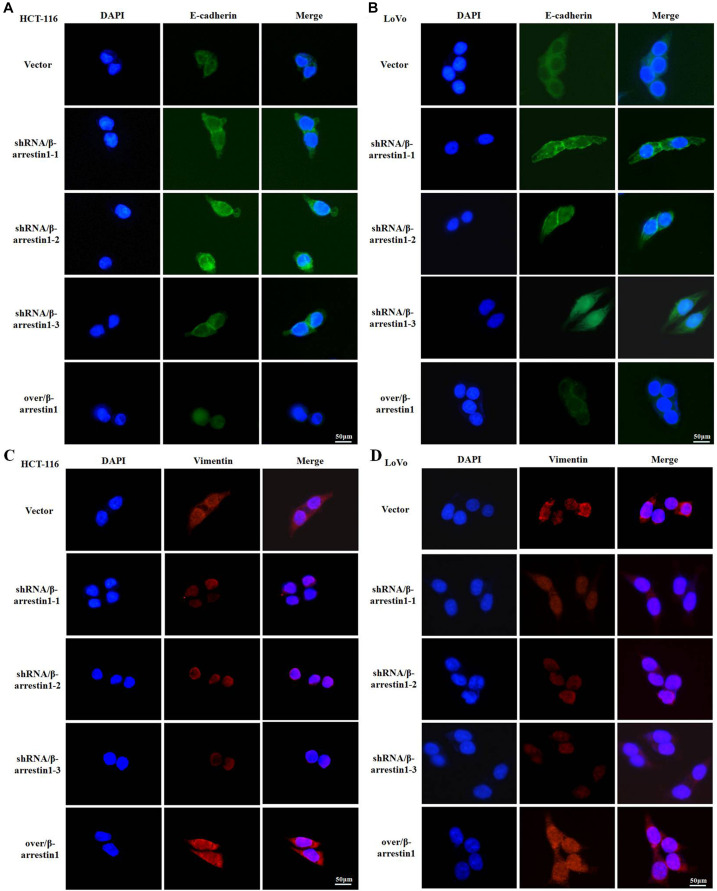
β-arrestin1 regulates the expression and cellular location of EMT markers. **(A–D)** Immunofluorescence staining of E-cadherin and Vimentin (two typical EMT markers) in HCT-116 or LoVo cells transfected with shRNA/β-arrestin1 or over/β-arrestin1 lentivirus. EMT, epithelial-to-mesenchymal transition.

Since E-cadherin and Vimentin are the most characteristic proteins of EMT, we further detected their expression by immunofluorescence. The results demonstrated that, compared with the control groups, the expression of E-cadherin was increased in the shRNA/β-arrestin1 group, but reduced in the over/β-arrestin1 group. However, in contrast to the control groups, the expression of Vimentin was downregulated in the shRNA/β-arrestin1 group, but upregulated in the over/β-arrestin1 group (Figures 3A–D and Supplementary Figure 1). Moreover, the immunofluorescence dual staining experiment for β-arrestin and E-cadherin (or Vimentin) in HCT-116 cells transfected with shRNA/β-arrestin1 or over/β-arrestin1 lentivirus also demonstrated that, the up-regulation of β-arrestin1 reduced the expression of E-cadherin but enhanced the expression of Vimentin, while the down-regulation of β-arrestin1 did the opposite (Supplementary Figure 2).

### β-Arrestin1 Promotes the Migration of CRC Cells

**FIGURE 4:**
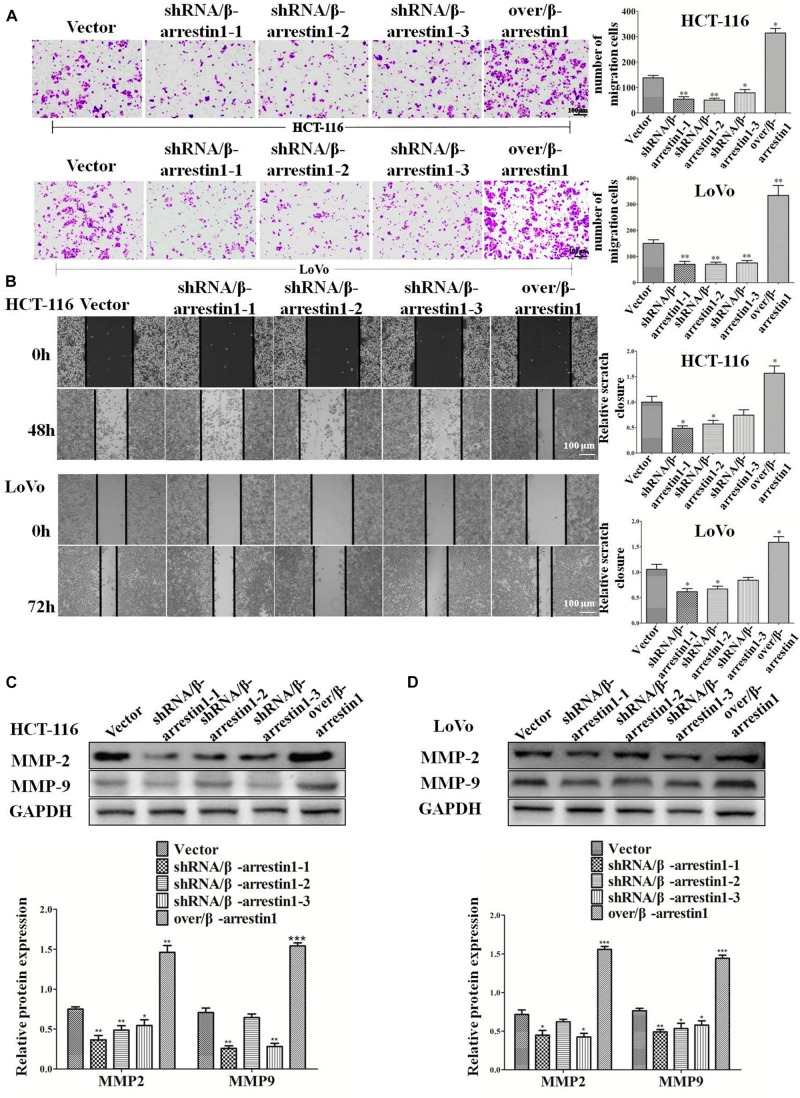
β-arrestin1 promotes the migration of CRC cells. **(A)** HCT-116 or LoVo cells transfected with shRNA/β-arrestin1 or over/β-arrestin1 lentivirus were used in the Transwell experiment. Typical images of cells migrating in a transwell migration chamber were captured and quantitatively measured. *P < 0.05; **P < 0.01 vs. HCT-116-vector or LoVo-vector cells. **(B)** shRNA/β-arrestin1 or over/β-arrestin1 cells were cultured to confluence at 4.5 × 10^5^ cells on 6-well plates. Representative images and a bar graph of the wound-healing assay are presented. The black line was used to mark the ranges of the scratches. *P < 0.05 vs. HCT-116-vector or LoVo-vector cells. **(C,D)** MMP2 and MMP9 were examined *via* western blotting. *P < 0.05; **P < 0.01; ***P < 0.001 vs. HCT-116-vector or LoVo-vector cells. CRC, colorectal cancer; shRNA, short hairpin RNA.

Epithelial-to-mesenchymal transition is the initial step of invasion and metastasis of cancer cells. The aforementioned results demonstrated that β-arrestin1 could regulate the EMT in CRC cells, and so further investigated the effect of β-arrestin1 on invasion and metastasis. Through Transwell and wound healing assays, the effects of β-arrestin1 on CRC cell migration was observed. According to the results of the Transwell analysis, it was revealed that shRNA/β-arrestin1 can markedly suppress the migration of HCT-116 or LoVo cells, but over/β-arrestin1 had the opposite effect (Figure 4A). In addition, we also demonstrated that shRNA/β-arrestin1 can markedly suppress the invasive ability of HCT-116 or LoVo cells, but over/β-arrestin1 had the opposite effect (Supplementary Figure 3). The results of the wound healing assays were similar to that of the Transwell migration assay (Figure 4B). Furthermore, we detected the expression levels of MMP2 and MMP9, which are closely associated with invasion and metastasis. Compared with the control group, the expression levels of MMP2 and MMP9 were downregulated in the shRNA/β-arrestin1 group, while over/β-arrestin1 could upregulate the expression levels of MMP2 and MMP9 in HCT-116 or LoVo cells (Figures 4C,D).

### β-Arrestin1 Activates the Wnt/β-Catenin Signaling Pathway in CRC

**FIGURE 5:**
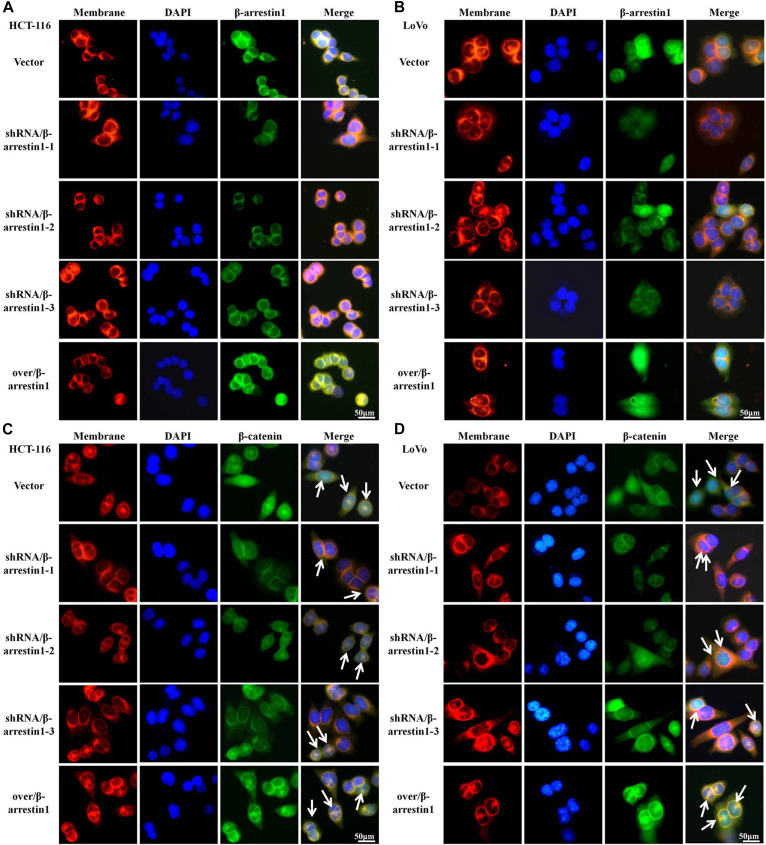
β-arrestin1 promotes the nucleus translocation of β-catenin protein in CRC cells. **(A,B)** Immunofluorescence staining of β-arrestin1 in shRNA/β-arrestin1 and over/β-arrrestin1 groups. **(C,D)** Immunofluorescence staining for β-catenin (green), nucleus (blue) and membrane (red) using an inverted fluorescence microscope (magnification, ×400). CRC, colorectal cancer.

A series of studies indicated that Wnt/β-catenin was participated in tumor invasion and metastasis (Duan et al., 2016). When Wnt/β-catenin signaling is activated, the translocation of β-catenin into the nucleus from the cytoplasm is increased (Clevers and Nusse, 2012). To interpret the function of β-arrestin1 on the expression and location of β-catenin, we performed immunofluorescence assays to examine the expression of β-arrestin1 in the shRNA/β-arrestin1 and over/β-arrestin1 groups. β-arrestin1 was expressed at low levels in the shRNA/β-arrestin1 group, and highly expressed in the over/β-arrestin1 group (Figures 5A,B). The quantitative results were also confirmed by reverse transcription quantitative PCR in Supplementary Figure 4. Then, our work further revealed that shRNA/β-arrestin1 decreased the nucleus translocation of β-catenin in CRC cells, but over/β-arrestin1 increased the expression of β-catenin (Figures 5C,D).

**FIGURE 6:**
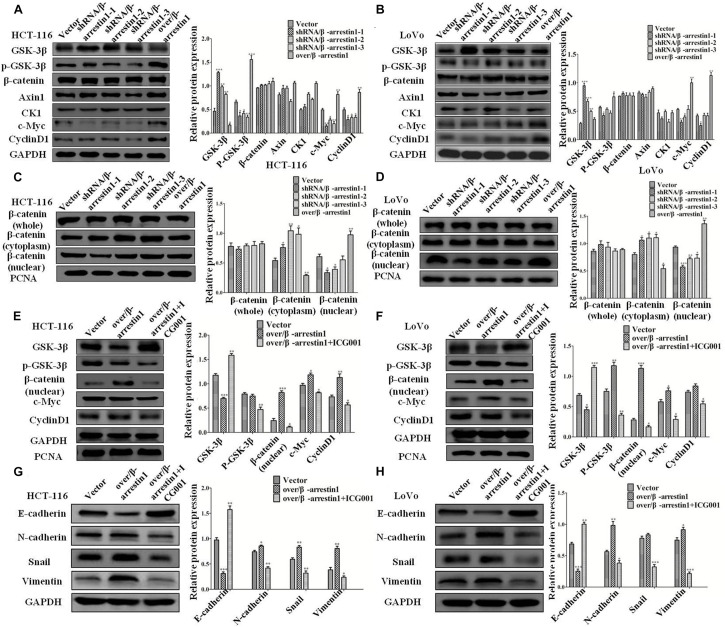
β-arrestin1 regulates the Wnt/β-catenin signal pathway in CRC cells. **(A,B)** Western blotting detected the protein levels of GSK-3β, p-GSK-3β, Axin1, CK1, β-catenin, c-Myc and CyclinD1 in shRNA/β-arrestin1 and over/β-arrestin1 groups. *P < 0.05; **P < 0.01; ***P < 0.001 vs. HCT-116-vector or LoVo-vector cells. **(C,D)** The protein expression of β-catenin in whole or cell extracts of different cellular compartments from HCT-116 or LoVo cells. *P < 0.05; **P < 0.01; ***P < 0.001 vs. HCT-116-vector or LoVo-vector cells. **(E,F)** β-catenin inhibitor ICG001 (10 μM) was added to the over/β-arrestin1 group, and then cultured for 24 h. The associated proteins of the Wnt signaling pathway were examined *via* western blotting. *P < 0.05; **P < 0.01 vs. HCT-116-vector or LoVo-vector cells. **(G,H)** Western blotting detected the protein expression levels of epithelial or mesenchymal markers after using ICG001. *P < 0.05; **P < 0.01; ***P < 0.001 vs. HCT-116-vector or LoVo-vector cells. CRC, colorectal cancer.

To understand the mechanism of β-arrestin1 on EMT in detail, this study examined the function of β-arrestin1 in modulating the expression levels of GSK-3β, p-GSK-3β, Axin1, CK1, β-catenin, c-Myc and CyclinD1, which are related to Wnt signaling and the downstream proteins of β-catenin. From the data in the report, it was showed that shRNA/β-arrestin1 promoted the protein expression of GSK-3β, while inhibiting the protein expression of p-GSK-3β, c-Myc and CyclinD1, yet over/β-arrestin1 had a promoting effect in HCT-116 or LoVo cells (Figures 6A,B). Furthermore, β-arrestin1 had no obvious change in the whole β-catenin expression. Next, we detected the expression of β-catenin in the cytoplasm and nucleus. As presented in Figures 6C,D, expression of β-catenin in the cytoplasm increased, and expression in the nucleus decreased in the shRNA/β-arrestin1 group, while over/β-arrestin1 had the opposite result. Next, β-arrestin1-overexpressing HCT-116 or LoVo cells were treated with or without ICG001 for 24 h, and the protein levels of Wnt/β-catenin signaling, including GSK-3β, p-GSK-3β, β-catenin, c-Myc and CyclinD1, were detected *via* western blotting. The results indicated that the expression levels of p-GSK-3β, β-catenin, c-Myc and CyclinD1 were all upregulated in the over/β-arrestin1 group, except for GSK-3β expression, yet this effect was reversed by blocking Wnt/β-catenin signaling with ICG001 (Figures 6E,F). Further study showed that, in over/β-arrestin1 group, the high level of β-catenin in the nucleus decreased after treatment with ICG001 (Supplementary Figure 4). The expression level of E-cadherin was upregulated, but the expression levels of N-cadherin, Snail and Vimentin were downregulated using ICG001 (Figures 6G,H). Taken together, these data indicated that β-arrestin1 exerts its effects following activation of the Wnt/β-catenin signaling pathway in CRC cells.

### β-Arrestin1 Regulates CRC Metastasis *in vivo*

**FIGURE 7:**
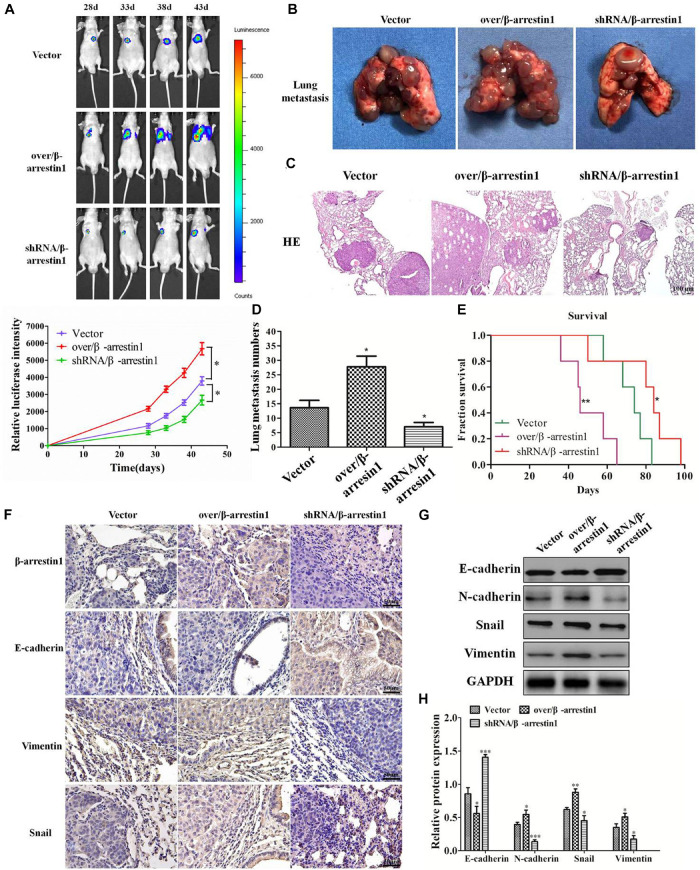
β-arrestin1 regulates CRC EMT and metastasis *in vivo*. **(A)** Fluorescence intensity was evaluated in eight mice of each group at days 28, 33, 38 and 43 after tail vein injection. *P < 0.05 vs. HCT-116-vector group. **(B–E)** The lung metastasis tissues were excised after day 43, and the number of metastatic lesions was counted. *P < 0.05 vs. HCT-116-vector group. The survival curve of animals injected with HCT-116/luc or shRNA/β-arrestin1-HCT-116/luc or over/β-arrestin1-HCT-116/luc cells. *P < 0.05; **P < 0.01 vs. HCT-116-vector group. Representative hematoxylin and eosin staining of tumor cytostructure and cytomorphology analysis. **(F)** Immunohistochemistry detected the expression levels of β-arrestin1, E-cadherin, Vimentin and Snail in three groups. **(G,H)** The protein expression levels of E-cadherin, N-cadherin, Snail and Vimentin were examined *via* western blotting. *P < 0.05; **P < 0.01, ***P < 0.001 vs. HCT-116-vector group.

In order to detect the function of β-arrestin1 in tumor metastasis *in vivo*, we used a lung metastatic tumor model of fluorescence-labeled CRC cell line, including HCT-116/luc, shRNA/β-arrestin1-HCT-116/luc and over/β-arrestin1-HCT-116/luc cells. Lung metastasis was observed on days 28, 33, 38, and 43. As demonstrated in Figure 7A, the shRNA/β-arrestin1 group showed less lung metastasis than the controls, while the over/β-arrestin1 group showed the most lung metastasis in three groups. Then, the amount of lung metastasis in the shRNA/β-arrestin1 and over/β-arrestin1 HCT-116 cells in the metastatic tumor model was assessed, and fewer lung metastatic sites were observed in the lungs of the shRNA/β-arrestin1 group. Among the three groups, the shRNA/β-arrestin1 group showed the best results with regard to extending the overall survival times. After that, the metastatic lesions were excised and examined by HE staining. According to the imaging results, it was revealed that more metastatic lesions were observed in the lungs with over/β-arrestin1, and the metastatic lesions markedly decreased in the shRNA/β-arrestin1 group (Figures 7B–E). Immunohistochemistry was applied to estimate the expression levels of β-arrestin1, E-cadherin, Vimentin and Snail. Compared with the controls, shRNA/β-arrestin1 group presented a higher expression level of E-cadherin, while lower expression level of Vimentin, Snail and β-arrestin1; however, the over/β-arrestin1 group had the opposite result (Figure 7F). The results of the western blot assay were consistent with the immunohistochemistry (Figures 7G–H). In summary, the results of this paper showed that β-arrestin1 was able to promote the lung metastasis of CRC cells through regulating the EMT process *in vivo*.

## Discussion

In recent years, accumulating evidence has indicated that β-arrestin1 acts important roles in CRC progression, but its detailed mechanism in CRC remains unclear. In our study, the data showed that β-arrestin1 facilitated the metastasis of CRC cells through activating the Wnt/β-catenin signal pathway and initiating the EMT process. In details, β-arrestin1 promotes the translocation of β-catenin protein into the nucleus, activates the β-catenin signaling pathway, and further triggers the initiation of EMT, leading to CRC progression.

β-arrestins, as ubiquitously scaffolding proteins, involve in regulating cancer cell phenotypes, including cell proliferation, migration, invasion and metastasis (Buchanan et al., 2006; Dasgupta et al., 2011; Enslen et al., 2014; Purayil et al., 2015). In which, β-arrestin1 has been reported to be responsible for EMT in tumor cells through a variety of signaling molecules. However, the roles and mechanisms of β-arrestin1 in CRC have not yet been elucidated (Pillai et al., 2015; Duan et al., 2016).

Epithelial-to-mesenchymal transition is a crucial step for tumor cells to obtain invasive ability, which is essential in promoting tumor invasion and metastasis (Banyard and Bielenberg, 2015). Activation of the mesenchymal phenotype and suppression of the epithelial phenotype are the results of changes in gene expression, such as Snail, Twist and zinc-finger E-box-binding (ZEB) transcription factors. When these transcription factors are abnormally expressed, EMT will be initiated (Peinado et al., 2007). In order to observe the role of β-arrestin1 in CRC, we first observed the effect of β-arrestin1 on EMT in CRC cells. The data revealed that silencing of β-arrestin1 inhibited the EMT process in CRC cells, while overexpression of β-arrestin1 significantly promoted the EMT process in CRC cells, which were visualized by the downregulation of E-cadherin and upregulation of Snail, N-cadherin and Vimentin in CRC cells. The results demonstrated that β-arrestin1 potentially exerts a vital role in promoting EMT and facilitating CRC metastasis.

In addition, our results demonstrated the effect of β-arrestin1 on CRC cell migration. The data indicated that silencing of β-arrestin1 expression in CRC cells significantly inhibited cell migration, while overexpression of β-arrestin1 had an opposite effect. Therefore, the aforementioned *in vitro* results revealed that β-arrestin1 can promote CRC cell migration partly through initiating EMT, which are similar to the results of previous studies that have focused on the important roles of β-arrestin1 in prostate, ovarian and non-small cell lung cancer (Peinado et al., 2007; Laura et al., 2009; Zecchini et al., 2014; Pillai et al., 2015).

Then, we considered the potential mechanism underlying the way in which β-arrestin1 regulates the EMT and metastasis in CRC. Wnt/β-catenin signaling promotes carcinogenesis and progression in multiple types of cancer *via* activating β-catenin to trigger the transcription of its downstream target genes (Lu et al., 2014; Rath et al., 2015; Zhou et al., 2016). The interaction between β-arrestin1 and β-catenin increases β-catenin target gene expression, which is responsible for cell migration, invasion and the EMT (Banyard and Bielenberg, 2015). Therefore, our study further investigated the effect of β-arrestin1 on the β-catenin signaling pathway. The data demonstrated that overexpression of β-arrestin1 could increase the accumulation of β-catenin in the nucleus, and cause a reduction in the cytoplasm, but silencing of β-arrestin1 had the opposite effect (Bryja et al., 2007; Rosanò et al., 2013). Blocking the β-catenin signaling pathway would greatly decrease the promoting effect of β-arrestin1 on EMT in CRC, suggesting the important promoting effect of β-arrestin1 on EMT partly through the β-catenin signaling pathway in CRC progression.

In summary, the results of our study demonstrated the significant role of β-arrestin1 in the regulation of EMT in CRC cells, and β-arrestin1-mediated Wnt/β-catenin signal pathway may account for the potential mechanism of CRC metastasis.

## Data Availability Statement

The original contributions presented in the study are included in the article/Supplementary Material, further inquiries can be directed to the corresponding author/s.

## Ethics Statement

The studies involving human participants were reviewed and approved by the Ethics Committee of Shanghai University of Traditional Chinese Medicine. The patients/participants provided their written informed consent to participate in this study. The animal study was reviewed and approved by Animal Care and Use Committee at Shanghai University of Traditional Chinese Medicine.

## Author Contributions

QL and QJ designed the research and prepared the manuscript. QS, ZH, XW, LZ, and LY performed the experiments. QJ, QS, QL, NL, and HS analyzed the data. QL, QJ, JC, and YW conceived and supervised the project. All authors read and approved the final manuscript.

## Conflict of Interest

The authors declare that the research was conducted in the absence of any commercial or financial relationships that could be construed as a potential conflict of interest.

## Supplementary Material

Supplementary Figure 1 The mRNA and protein expression of β-arrestin1 in different groups of Figures 5A,B was validated by reverse transcription quantitative PCR and western bot quantitation. ^∗^*P* < 0.05; ^∗∗^*P* < 0.01 vs. HCT-116-vector or LoVo-vector cells.

Supplementary Figure 2 Immunofluorescence dual staining experiment for β-arrestin and E-cadherin (or Vimentin) in HCT-116 or LoVo cells transfected with shRNA/β-arrestin1 or over/β-arrestin1 lentivirus. shRNA, short hairpin RNA.

Supplementary Figure 3 β-arrestin1 promotes the invasion of CRC cells. HCT-116 or LoVo cells transfected with shRNA/β-arrestin1 or over/β-arrestin1 lentivirus were used in the Transwell experiment. Typical images of cells invaded and migrated in a transwell chamber were captured and quantitatively measured. ^∗^*P* < 0.05; ^∗∗^*P* < 0.01; ^∗∗∗^*P* < 0.001 vs. HCT-116-vector or LoVo-vector cells. CRC, colorectal cancer.

Supplementary Figure 4 The mRNA and protein expression of β-arrestin1 in different groups of Figures 5A,B was validated by reverse transcription quantitative PCR. ^∗^*P* < 0.05; ^∗∗^*P* < 0.01 vs. HCT-116-vector or LoVo-vector cells.

## Abbreviations

ATCC: American Type Culture Collection
CRC: colorectal cancer
EMT: epithelial mesenchymal transition
KEGG: Kyoto Encyclopedia of Genes and Genomes
GEO: Gene Expression Omnibus
GO: Gene Ontology
RT-qPCR: reverse transcription-quantitative PCR
HE: hematoxylin and eosin
FBS: fetal bovine serum
GPCR: G protein coupled receptor
TGF-β: transforming growth factor-β
PI3K: phosphatidylinositol 3-kinase
MAPK: mitogen-activated protein kinase
Wnt: Wingless/integration-1
PGE2: prostaglandin E2
EGFR: epidermal growth factor receptor.
