# Supplementary figures and images for: β-Arrestin1 Promotes Colorectal Cancer Metastasis Through GSK-3β/β-Catenin Signaling- Mediated Epithelial-to-Mesenchymal Transition
Source: Front Cell Dev Biol. 2021 Apr 28;9:650067. doi: 10.3389/fcell.2021.650067 (PMC8114940; doi:10.3389/fcell.2021.650067)

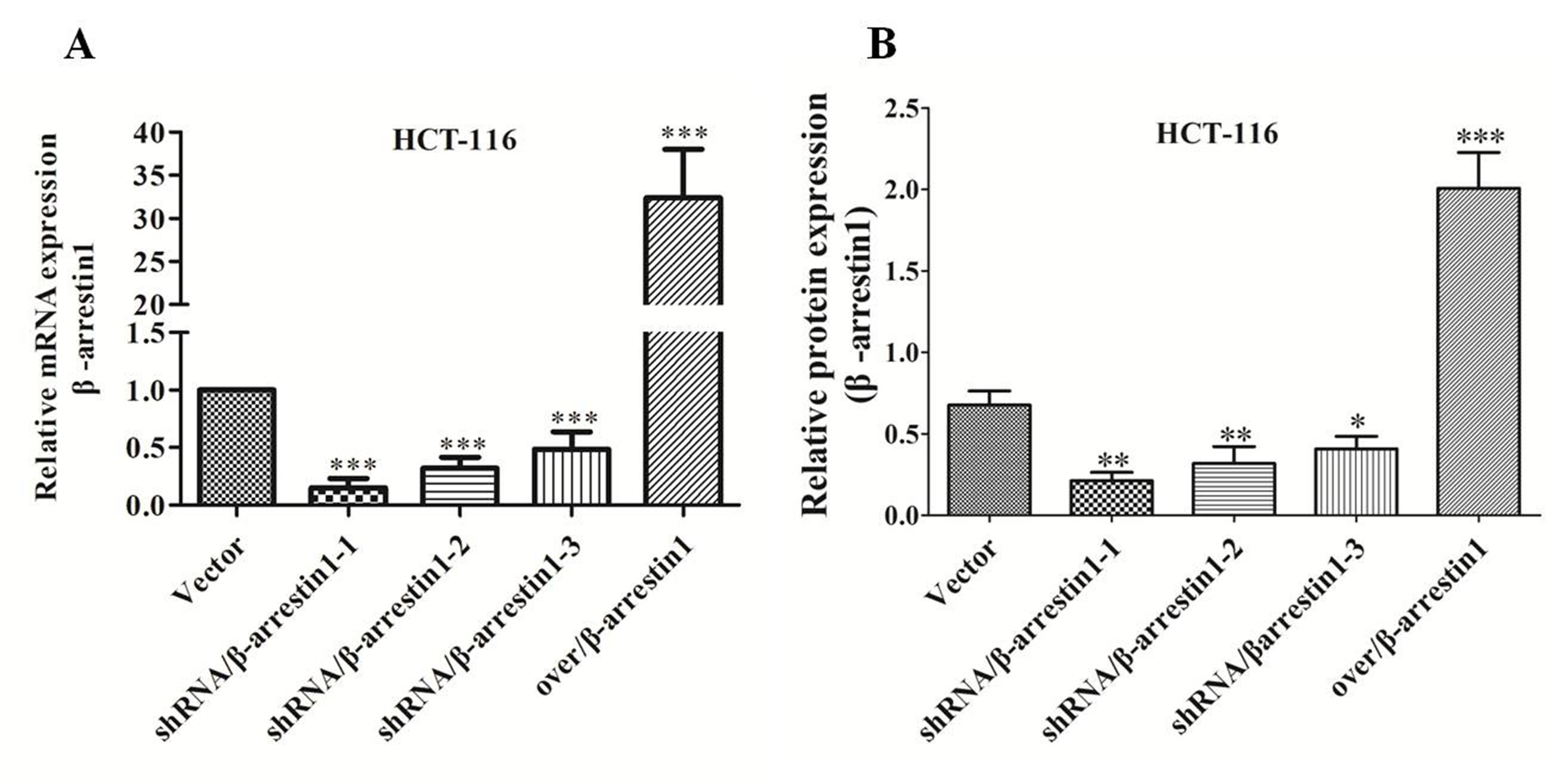

Supplement: Supplementary Figure 1 — The mRNA and protein expression of β-arrestin1 in different groups of Figures 5A,B was validated by reverse transcription quantitative PCR and western bot quantitation. ∗P < 0.05; ∗∗P < 0.01 vs. HCT-116-vector or LoVo-vector cells. [file Image_1.JPEG]

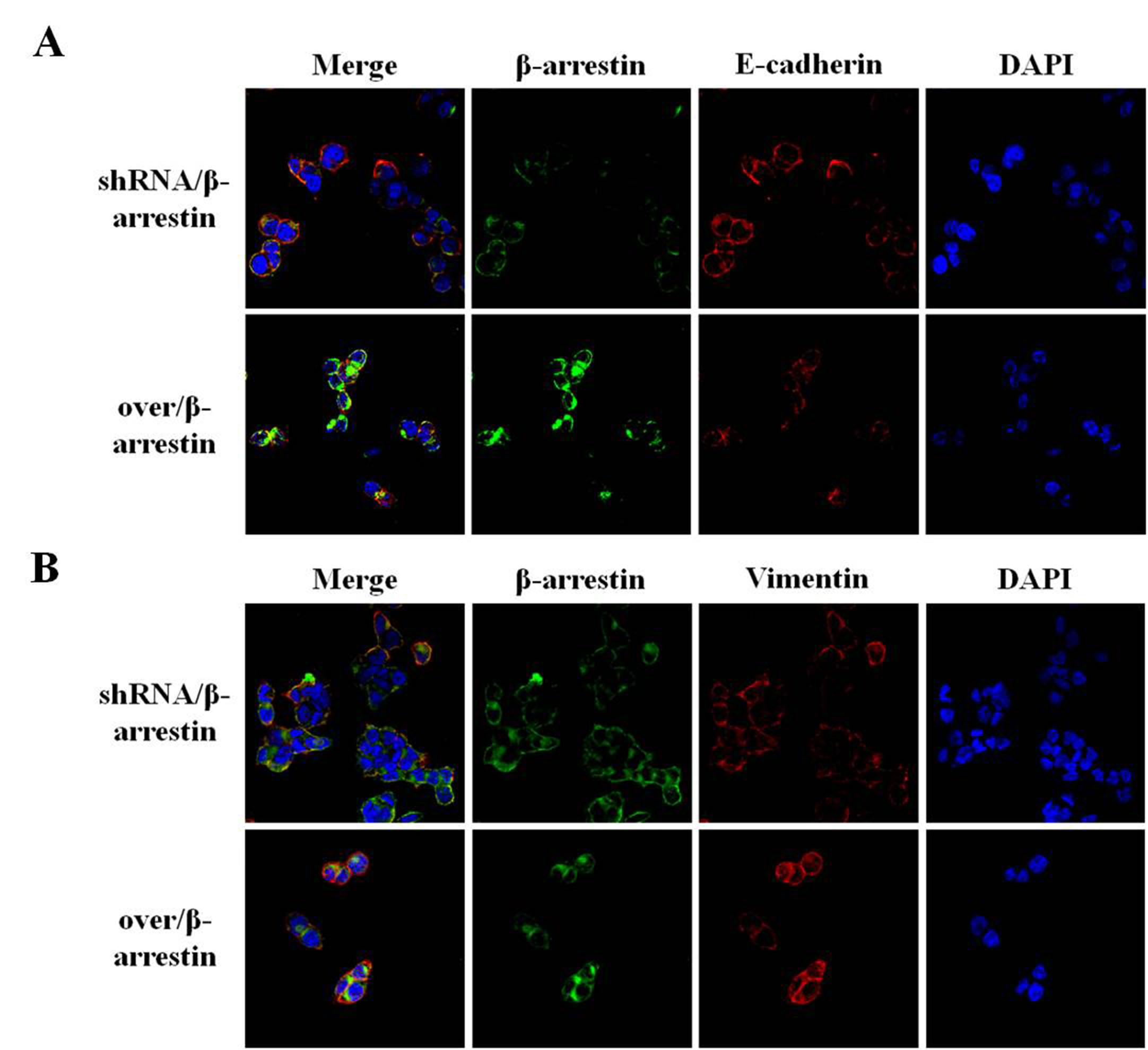

Supplement: Supplementary Figure 2 — Immunofluorescence dual staining experiment for β-arrestin and E-cadherin (or Vimentin) in HCT-116 or LoVo cells transfected with shRNA/β-arrestin1 or over/β-arrestin1 lentivirus. shRNA, short hairpin RNA. [file Image_2.JPEG]

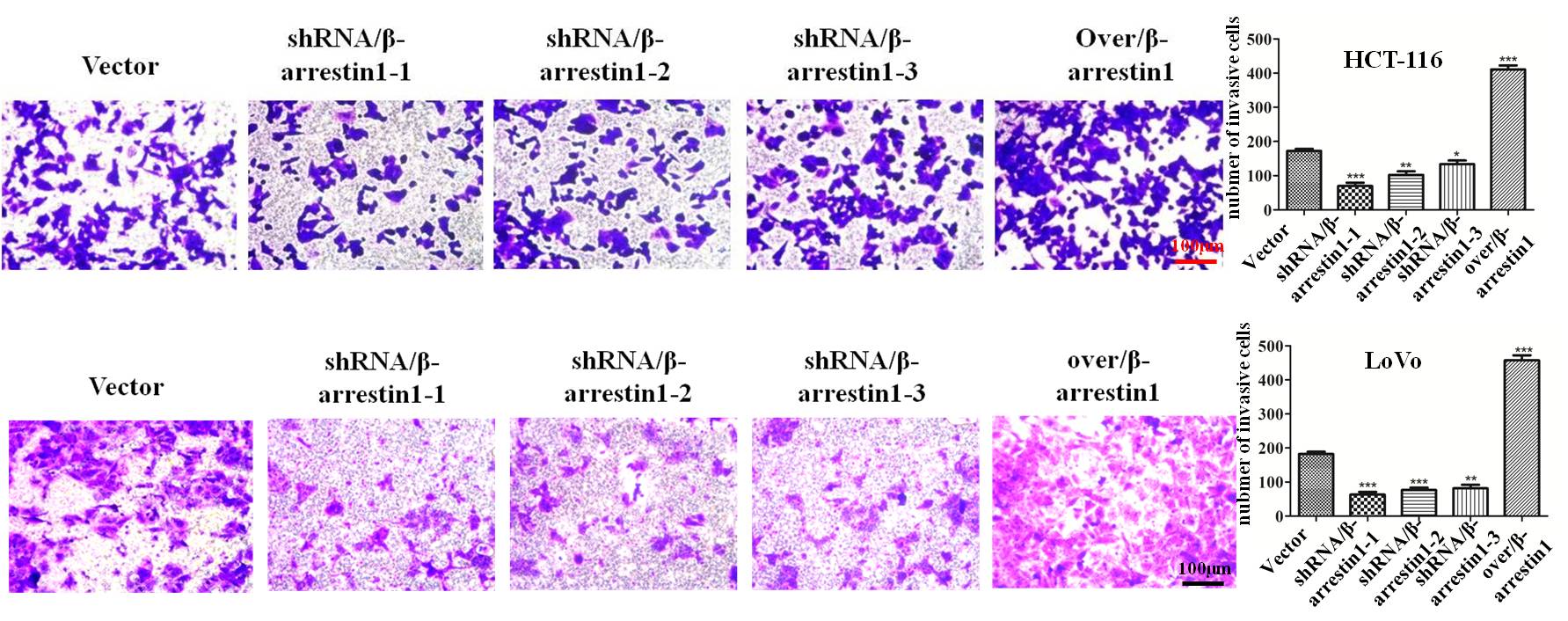

Supplement: Supplementary Figure 3 — β-arrestin1 promotes the invasion of CRC cells. HCT-116 or LoVo cells transfected with shRNA/β-arrestin1 or over/β-arrestin1 lentivirus were used in the Transwell experiment. Typical images of cells invaded and migrated in a transwell chamber were captured and quantitatively measured. ∗P < 0.05; ∗∗P < 0.01; ∗∗∗P < 0.001 vs. HCT-116-vector or LoVo-vector cells. CRC, colorectal cancer. [file Image_3.JPEG]

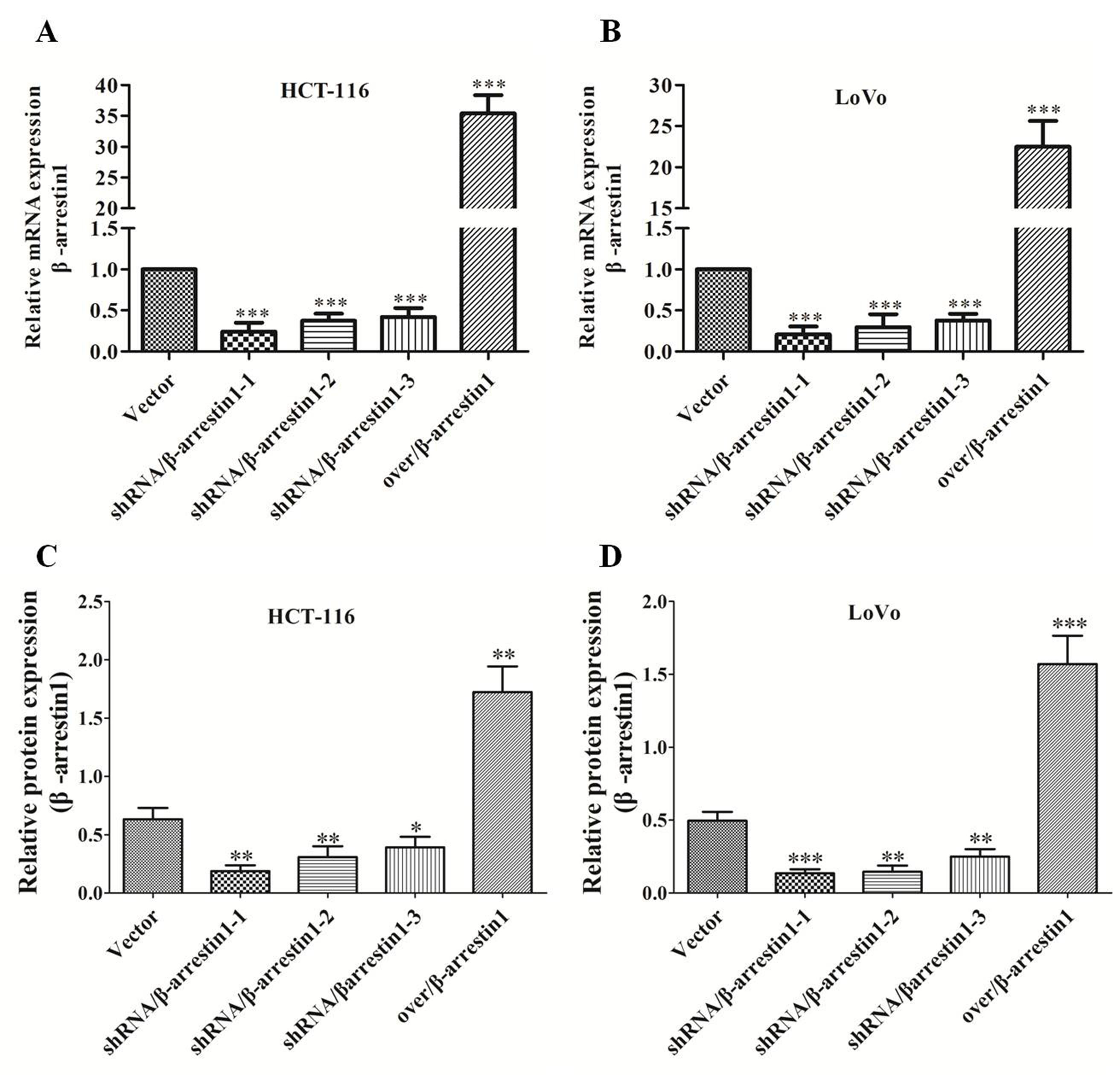

Supplement: Supplementary Figure 4 — The mRNA and protein expression of β-arrestin1 in different groups of Figures 5A,B was validated by reverse transcription quantitative PCR. ∗P < 0.05; ∗∗P < 0.01 vs. HCT-116-vector or LoVo-vector cells. [file Image_4.JPEG]
